# TFIIB-Related Factor 2 Over Expression Is a Prognosis Marker for Early-Stage Non-Small Cell Lung Cancer Correlated with Tumor Angiogenesis

**DOI:** 10.1371/journal.pone.0088032

**Published:** 2014-02-11

**Authors:** Ming Lu, Hui Tian, Weiming Yue, Lin Li, Shuhai Li, Lei Qi, Wensi Hu, Cun Gao, Libo Si

**Affiliations:** Department of Thoracic Surgery, Qilu Hospital, Shandong University, Jinan, P. R. China; University of Texas MD Anderson Cancer Center, United States of America

## Abstract

**Background:**

The aim of this study was to examine BRF2 expression in patients with non-small cell lung cancer (NSCLC) and explore the relationship of BRF2 protein with clinicopathologic factors, tumor angiogenesis and prognosis.

**Methods:**

Both BRF2 protein and intratumoral microvessels were examined by immunohistochemical staining in 107 non-small cell lung cancer patients. Intratumoral m icrovessel density (MVD) was measured by counting CD-34 positive immunostained endothelial cells. Western blot and RT-PCR analyses were utilized to investigate the BRF2 expression status in tissues

**Results:**

A notably higher level of BRF2 expression was found in NSCLC tissues at protein levels. In addition, univariate and multivariate analysis demonstrated that BRF2 protein over-expression and high MVD were significantly associated with tumor relapse. Although BRF2 overexpression and high MVD indicated poor 5-year overall survival (p = 0.004 and p = 0.019, respectively), multivariate analysis demonstrated that only BRF2 overexpression was an independent prognostic factor for unfavorable overall survival (P = 0.021).

**Conclusions:**

BRF2 is a promising biomarker to identify individuals with poor prognostic potential and a possible target for anti-angiogenic therapy for patients with early-stage NSCLC.

## Introduction

Lung cancer is the most common cause of cancer death for both men and women worldwide. Every year, there are 1.35 million new lung cancer cases in the world [1]. Non-small cell lung cancer (NSCLC) accounts for approximately 75–80% of cases of lung cancer [2]–[3]. Despite the advances in early detection, radical cure operation, and multimodal therapeutic modalities in the past decades, the overall 5-year survival rate of lung cancer is only 15% [4]. 30–40% of stage I patients relapse after surgical resection [5]. Moreover, prospective randomized data showed that adjuvant chemotherapy in stage IB has not achieved a significant survival benefit and even a detrimental effect was observed in stage IA [6]. Therefore, there is an urgent need to identify novel biomarkers that will help select the patients with high chance of lung cancer recurrence and provide better prognosis and individualization treatment.

RNA polymerase (pol) III is responsible for the transcription of small, less than 300 nucleotides, untranslated RNAs including microRNAs (miRNAs) [7]–[8]. Since their discovery, miRNAs have been implicated in the pathogenesis of various human cancers, and miRNA expression profiling of human tumors has specific signatures associated with the diagnosis, staging, progression, prognosis and response to treatment [9]. Accurate transcription by RNA pol III requires TFIIIB, while BRF2 (TFIIB-related factor 2) is a component of TFIIIB. The regulation of pol III is integral to the growth control functions of RB, P53 and c-Myc, and TFIIIB activity is strictly regulated by Maf1, chemopreventative agents, oncogenes and tumor suppressors [10]–[15]. BRF2 has been shown to be highly overexpressed in a variety of cancers including gastric, kidney and melanoma cancers [16]–[17]. Recently, Lockwood et al. reported that overexpression of BRF2 could drive the expression of RNA pol III transcripts, contributing to squamous cell carcinoma tumorigenesis, and BRF2 has been identified as a novel lineage-specific oncogene in lung squamous cell carcinoma [18]. However, to our knowledge, the expression of BRF2 and its correlation with clinicopathologic factors and prognosis in surgically resected NSCLC have not been investigated.

In this study, we employed immunohistochemical method to examine BRF2 protein expression in clinical NSCLC samples, and we analyzed the relationships of BRF2 expression with variable clinicopathologic features and patient prognosis. Moreover, we assessed the independent prognostic factors that affect long-term survival of NSCLC patients.

## Materials and Methods

### Ethics Statement

The study protocol was approved by Ethics Boards of Qilu Hospital, and tissue specimen acquisition was carried out in accordance with the institutional guidelines. All subjects signed written informed consent, and this consent procedure was approved by Ethics Boards of Qilu Hospital.

### Patients

Samples were obtained from 107 patients who were diagnosed with NSCLC between Jan. 2004 and Oct. 2006 at the Department of Thoracic Surgery, Qilu Hospital, and treated with pulmonary lobectomy plus regional lymph node dissection.

All patients had no preoperative radiotherapy or chemotherapy, and had no distant metastases. The data on their clinicopathologic features and follow-up were complete. 46 cases of adjacent tissue samples were taken from about 0.5 cm away from the outer edge of the lung tumor tissues, and the other 32 cases were taken more than 5 cm from the tumor margin of normal lung tissues as negative controls. For RT-PCR and Western blotting analysis, 12 matched pairs of tumors tissue and adjacent noncancerous tissue samples were obtained from pulmonary lobectomy specimens of patients diagnosed with NSCLC immediately after surgery between Sep 2013 and Oct 2013 in our department, and stored at −80°C.

For all patients, histological type and grade of cancer cell differentiation were reevaluated and determined by the classification system of the World Health Organization modified in 2004, and postsurgical pathological staging was determined based on the international staging system.

### Immunohistochemistry

All specimens were collected during the surgery, fixed by 10% formalin and embedded in paraffin. The tissues were cut as 4 µm serial sections, and then deparaffinized using xylene and rehydrated through an ethanol series to water. High-temperature antigen retrieval was carried out in citrate buffer for 25 min in a microwave oven. Then the endogenous peroxidase enzyme activity was blocked using 3% H_2_O_2_ in methanol for 20 min at room temperature. The slides were then incubated with primary rabbit anti-BRF2 polyclonal antibody (Abcam) and rabbit anti-CD34 monoclonal antibody (Santa Cruz Biotechnology) overnight at 4°C in a high humidity chamber, followed by incubation for 30 min at 37°C with biotinylated secondary antibodies and streptavidin-peroxidase complex. Finally, a 3,30-diaminobenzidine solution was added, and the slides were counterstained with hematoxylin and mounted with neutral balsam. For negative controls, sections were incubated with PBS instead of the primary antibodies.

### Evaluation of BRF2 protein expression and microvessel density

For BRF2 staining, the area within the diagnostic area was scored by three independent observers and a reproducible semi-quantitative method that considered both staining intensity (0, negative; 1, weak; 2, moderate and 3, strong) and the percentage of positively stained cells (0, 0–5%; 1, 6–25%; 2, 26–50%; 3, 51–75%; 4, >76%) was adopted [19]. For evaluation of the positive staining of BRF2, at least 3 sections or areas from each sample should be scored. Conflicting scores were resolved by choosing the value consistent between two observers or the average of the scores.

For intratumoral microvessel density (MVD), microvessels were recorded by counting CD34 positively stained endothelial cells as descripted previously [20]. The microvessel count (MVC) was determined independently by two pathologists in each case. Three representative areas of dense neovascularization were selected under low microscope power (×10 objective lens and ×10 ocular lens) and then vessels were counted at higher magnification (×20 objective lens and ×20 ocular lens). The average of the counts in three fields was recorded. Large vessels with thick, muscular walls were excluded.

The cutoff value for high and low expression was determined based on a heterogeneity value measured through log-rank statistical analysis with respect to overall survival [21]. The staining index score 4 was chosen as cutoff point for discrimination between BRF2 low and high expression. And the staining index score≥4 defined tumors with high BRF2 expression, and the staining index score<4 indicated low BRF2 expression. Tumors with microvessels ≥42 were classified as high MVD, while tumors with microvessels <42 were classified as low MVD.

### Real-time PCR (RT-PCR)

Surgical specimens were processed immediately after operation. Total RNAs were extracted from tissues by using Trizol reagent (Invitrogen, Carlsbad, USA) according to the manufacturer's protocol and treated with RQ1 RNase-free DNase (Promega) cDNA was synthesized. The expressions of BRF2 and HIF-1α were quantified by real-time polymerase chain reaction (PCR) using a Bio-Rad iQ5 real-time PCR system with EvaGreen Supermix (Bio-Rad, Hercules, USA) according to the instruction manual. The primer for GAPDH and BRF2 are as follows: GAPDH, forward,5′-AGGTCGGTGTGAACGGATTTG-3′,reverse,5′-TGTAGACCATGTAGTTGAGGTCA-3′;BRF2,forward,5′-GTGAAGCTCCTGGGACTGGAT-3′,reverse, 5′-GTATTTGGCTGGCACAGAAGG-3′; Results are mean±standard error mean (SEM) of 3 repeat experiments and GAPDH was used as a reference transcript.

### Western blotting analysis

The fresh tissues were washed three times with ice-cold phosphate-buffered saline (PBS) and lysed on ice in RIPA (radio immunoprecipitation assay) buffer (Cell Signaling Technology, Danvers, MA,USA) containing complete protease inhibitor cocktail (Roche Applied Science, Mannheim, Germany). Protein from tissues or cells were separated via SDS-PAGE and transferred to a PVDF membrane (GE healthcare, USA). Membranes were blocked with 5% fat-free milk in Tris-buffered saline containing 0.1% Tween-20 (TBST) for 1.5 h at room temperature, the membranes were then incubated overnight at 4°C with anti-BRF2(1∶1000,Abcam, Cambridge, MA, USA), or anti-GADPH (1∶1000, Abcam, Cambridge, MA, USA) antibodies. Followed by anti- rabbit horseradish peroxidase conjugated IgG, an ECL kit (GE healthcare, USA) was used for detection.

### Statistical analysis

Chi-square test was used to test the correlation between BRF2 expression and MVD, and the associations between BRF2 expression or MVD and clinicopathological factors. Kaplan–Meier method was used to calculate the survival curves, and log-rank test was used to compare the difference between the survivals of patient subgroups. Multivariate Cox regression analysis was used to identify significant independent prognostic factors. Differences between groups were considered significant for P value<0.05. All statistical analyses were performed with SPSS 13.0 statistical software (SPSS Inc., Chicago, IL, USA).

## Results

### BRF2 expression in NSCLC

**Figure 1:**
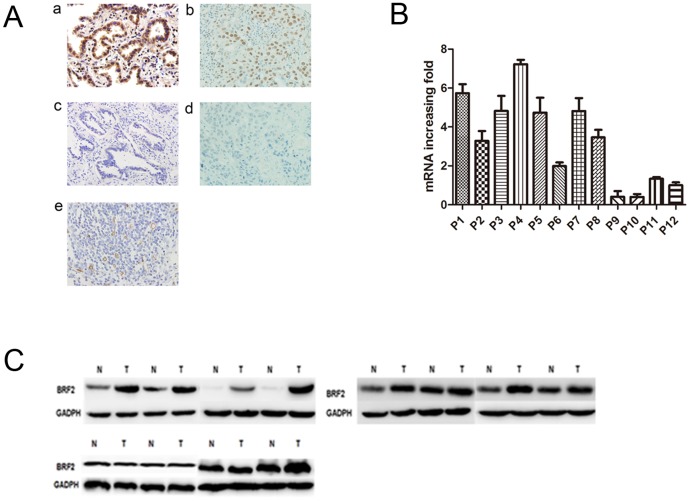
(A): The expression pattern of BRF2 in lung cancer tissues. (a) High BRF2 expression in lung adenocarcinoma. (b): High BRF2 expression in lung squamous cell carcinoma tissues. (c): Negative BRF2 expression in lung adenocarcinoma. (d): Negative BRF2 expression in lung squamous cell carcinoma tissues. (e): Intratumoral microvessels were stained as brown by the anti-CD34 monoclonal antibody in lung cancer tissues (magnification×400). (B): Quantitative real-time PCR analyses of BRF2 mRNA in twelve pairs of matched NSCLC and noncancerous tissues with GAPDH as a loading control in both panels. (C): Protein levels of BRF2 expression were evaluated by western blotting from paired noncancerous tissue and NSCLC.

We detected the expression of BRF2 protein in the normal lung tissues, adjacent non-tumor tissues and tumor tissues by immunohistochemistry. As shown in Fig. 1 A, diffuse nuclear staining of BRF2 protein at various intensities was observed in cancer cells, but BRF2 was barely detected in normal lung tissues. In addition, some staining was observed in the cytoplasm of cancer cells. However, we observed no statistically significant correlation between BRF2 protein expression and any clinicopathological features of NSCLC tissues (P>0.05, Table 1). The mean value of BRF2 expression in 107 NSCLC tissues was 55.14%, significantly higher than that in adjacent tissues and normal lung tissues (36.96%, and 34.38%, respectively, P = 0.034 Table 2).

**Table 1:** The correlation of clinicopathologic variables of NSCLC with BRF2 and MVD.

		BRF2 overexpression				MVD		
Variable		No. of patients	no	yes	p	Low	High	P
Age	≤65 years	43	21	22	0.498	17	26	0.453
	>65 years	64	27	37		30	34	
Gender	Male	51	20	31	0.263	22	29	0.875
	Female	56	28	28		25	31	
Smoking	No	53	24	29	0.931	27	26	0.147
	Yes	54	24	30		20	34	
Histology	Adeno	48	25	23	0.175	22	26	0.720
	Squamous	59	23	36		25	34	
Differentiation	Well	23	11	12	0.086	7	16	0.195
	Moderate	54	19	35		28	26	
	Poor	30	18	12		12	18	
Invasion depth	T1	47	23	24	0.453	27	20	0.013
	T2	60	25	35		20	40	

**Table 2:** The expression of BRF2 in lung cancer, adjacent lung cancer tissues and normal lung tissues.

Variable	n	Positive (n)	Positive rate (%)	χ2	P
Cancer tissue	107	59	55.14%	6.756	0.034
Adjacent tissue	46	17	36.96%		
Normal tissue	32	11	34.38%		

To investigate the status of BRF2 gene expression in NSCLC, we used Real-time PCR to measure the mRNA expression in 12 pairs of primary cancer tumors and adjacent noncancerous specimens. Compared with their adjacent noncancerous specimens, 8 of 12 NSCLC had up-regulated expression (Fig. 1B and Fig. 1 C). Consistently, western blot analysis showed that the 8 cases also had higher BRF2 protein expression than adjacent tissues.

### Correlation between MVD and clinicopathologic factors of NSCLC

Intratumoral MVD was quantified by counting CD34-positive endothelial cells in cancer tissues (Fig. 1A), and the staining intensity of MVD ranged broadly from 12 to 118 microvessels/200×magnification field. We found that MVD was significantly correlated with invasion depth (P = 0.013), but not with other clinicopathologic factors of NSCLC (P>0.05; Table 1).

### Univariate and multivariate survival analysis

**Figure 2:**
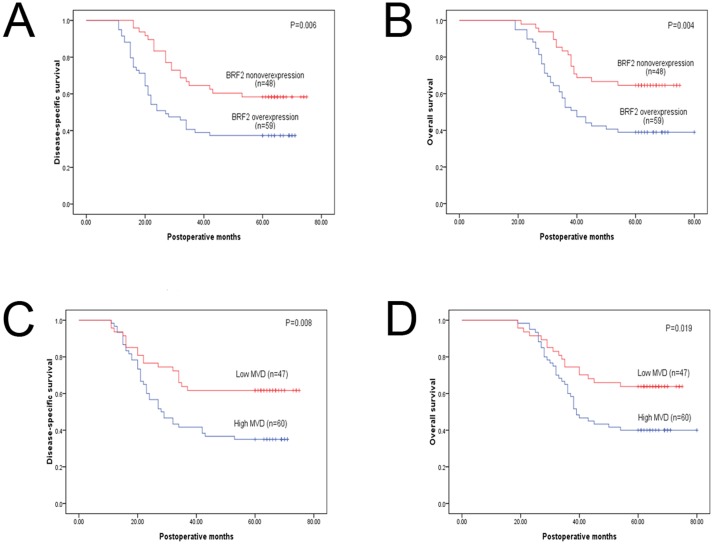
Kaplan–Meier curves of disease-free and overall survival stratified according to the status of BRF2 Protein expression and MVD.

Of the 107 patients, 53 (49.5%) cases died within 5 years after operation, and tumor relapse developed during follow-up in 57 (53.3%) patients. Kaplan-Meier analyses compared by the log-rank test were used to calculate the effect of the clinicopathologic factors on overall survival and disease-free survival. Univariate analysis demonstrated that high MVD (40.0% vs. 63.8%; P = 0.019) and BRF2 protein overexpression (39.0% vs.64.6%; P = 0.004) significantly predicted decreased overall 5-year survival. In addition, high MVD (65.0 vs. 38.3%; P = 0.008) and BRF2 protein overexpression (62.7% vs. 41.7%; P = 0.006) indicated a higher risk of recurrence (Fig. 2); Furthermore, multivariate analysis identified BRF2 overexpression (P = 0.036) and MVD (P = 0.034) as independent prognostic factors for progression-free survival. However, only BRF2 overexpression retained its significance as an independent prognostic factor for overall as well as progression-free survival (P = 0.021, Table 3 and Table 4).

**Table 3:** Univariate and multivariate analyses for disease-free survival.

	PFS Univariate analysis	PFS Multivariate analysis		
Variable	P value (log-rank test)	95.0% confidence interval	Exp (B)	P value
Gende (male vs female)	0.293	0.443–1.302	0.759	0.317
Age (≤60 and >60)	0.214	0.368 –1.125	0.644	0.122
Smoking (yes vs no)	0.471	0.514–1.513	0.882	0.648
Histology (Sq vs Ad)	0.315	0.456–1.316	0.775	0.345
T status (T2 vs T1)	0.760	0.588–1.819	1.034	0.908
Differentiation(Poorly vs Well and Moderate)	0.827	0.599–1.248	0.865	0.437
BRF2 protein (high vs low)	0.006	1.039–3.225	1.831	0.036
MVD (high vs low)	0.008	1.049–3.488	1.913	0.034

**Table 4:** Univariate and multivariate analyses for overall survival.

	OS Univariate analysis	OS Multivariate analysis		
Variable	P value (log-rank test)	95.0% confidence interval	Exp (B)	P value
Gender (male vs female)	0.122	0.356–1.101	0.626	0.104
Age (≤60 vs >60)	0.296	0.392–1.227	0.694	0.209
Smoking (yes vs no)	0.412	0.490–1.516	0.862	0.607
Histology (Sq vs Ad)	0.303	0.439–1.312	0.759	0.324
T status (T2vs T1)	0.640	0.625–2.003	1.119	0.706
Differentiation(Poorly vs Well and Moderate)	0.903	0.627–1.339	0.916	0.651
BRF2 protein (high vs low)	0.004	1.113–3.764	2.047	0.021
MVD (high vs low)	0.019	0.910–3.165	1.697	0.096

**Figure 3:**
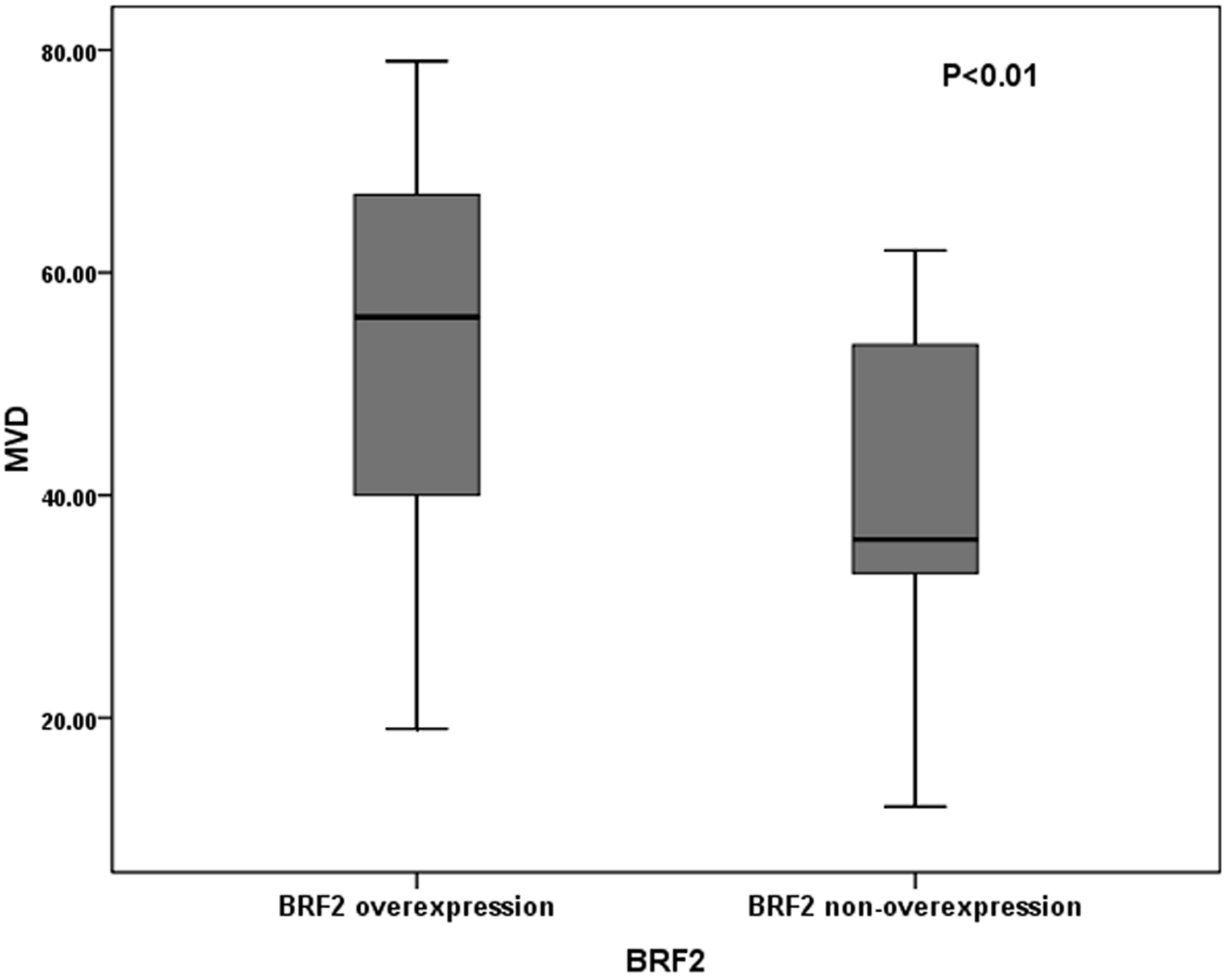
Intratumoral microvessel density (MVD) in relation to BRF2 protein immunoreactivity. Mann–WhitneyUtest demonstrated that tumors with BRF2 protein high expression showed significantly higher intratumoral MVD than tumors with BRF2 protein low expression (P<0.001).

To explore the correlation of BRF2 overexpression with MVD, we further examined the survival differences of patients stratified for low MVD and high MVD according to BRF2 protein expression status. For patients without BRF2 overexpression, we detected a highly significant inferior overall survival (OS) and Disease-free survival (PFS), respectively, in patients with high MVD compared with patients with low MVD (P = 0.003 for OS and P = 0.001 for PFS, Table 5). However, there were no significant differences in survival between low-MVD group and high-MVD group for patients with BRF2 overexpression (P>0.05, Table 5). And statistical analysis demonstrated that there was significantly more MVD in tumors with BRF2 protein high expression than that in those with BRF2 protein low expression (P<0.001, Mann–Whitney U test; Fig. 3).

**Table 5:** Survival differences stratified by low MVD and high MVD in patients with or without BRF2 overexpression.

	BRF2 overexpression			BRF2 nonoverexpression		
5-year survival	Low MVD	High MVD	p	Low MVD	High MVD	p
Overall	36.84%	40.00%	0.816	82.14%	40.00%	0.003
Disease-free	35.00%	38.46%	0.795	78.57%	30.00%	0.001

**Figure 4:**
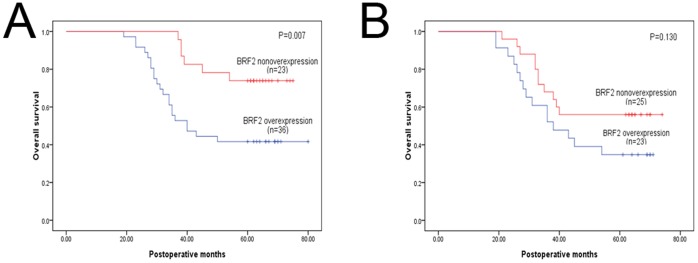
Kaplan-Meier survival curves of patients stratified according to histology type. (a): squamous cell carcinoma. (b): adenocarcinoma.

We further analyzed the prognostic significance of BRF2 protein in selective patient subgroups stratified according to histology type of NSCLC. Univariate analysis demonstrated that the overall 5-year survival rate of patients with BRF2 protein high expression was significantly lower than that of the remaining patients among squamous cell carcinoma, and we also found such a trend in adenocarcinoma, despite the statistical analysis does not make sense; (P = 0.007 and P = 0.130, respectly; Fig. 4).

## Discussion

The ability to proliferate uncontrollably is the dominant characteristic of many types of cancer cells. Evaluation of molecular prognostic factors is one important area of cancer research. BRF2 protein is encoded by a gene located on chromosome 8p12 [22]. Several studies have shown that BRF2 is overexpressed in several types of cancer [23] and suggest the oncogenic role of BRF2. However, few studies have investigated the expression and significance of BRF2 in NSCLC, especially for the prognosis of NSCLC.

In this study, our results showed that there was no significant correlation between BRF2 expression and the clinicopathological features of NSCLC (p>0.05). However, high MVD was significantly associated with T status in NSCLC, but not associated with age, gender, smoking, histology and differentiation. To a certain extent, T stage is a critical process of tumor development, and the difference in the correlation between MVD and clinicopathological features may reflect that the microvessel density is an important aspect of tumor development. However, we found no significant difference in BRF2 expression between lung adenocarcinoma and lung squamous cell carcinoma by immunohistochemical staining.

Notably, our results showed that BRF2 protein overexpression was common in early NSCLC tissues and significantly associated with increased angiogenic activity measured as intratumoral MVD, suggesting that BRF2 plays crucial role in NSCLC tumorigenesis by the induction or/and promotion of tumor angiogenesis. Although multiple growth factors have been shown to regulate angiogenesis and vascular development, little is known about the complex regulation mechanism of gene expression and translation [24]. Our results highlight the potential role of BRF2 in tumor angiogenesis.

Our survival analysis demonstrated that high MVD and BRF2 protein overexpression significantly predicted decreased overall 5-year survival and higher recurrence rate. Further analysis using the Cox regression model confirmed that BRF2 expression and MVD were independent factors in predicting progression-free survival for NSCLC patients, suggesting that BRF2 protein and MVD may be potential prognostic factors for the relapse of early-stage NSCLC patients. However, in multivariate analysis, only BRF2 expression could independently and significantly predict overall 5-year survival, despite the finding that high MVD was significantly associated with tumor recurrence. Our data indicated that BRF2 overexpression had an overwhelming influence on the OS and PFS, while MVD showed a significant effect on the OS and PFS only in the patients without BRF2 overexpression.

Taken together, our data support the assumption that BRF2 protein over-expression is common in early-stage NSCLC and significantly correlated with tumor angiogenesis and relapse. Moreover, BRF2 overexpression is an independent prognostic factor for NSCLC patients.
